# Second-generation Flagellin-rPAc Fusion Protein, KFD2-rPAc, Shows High Protective Efficacy against Dental Caries with Low Potential Side Effects

**DOI:** 10.1038/s41598-017-10247-8

**Published:** 2017-09-11

**Authors:** Jingyi Yang, Ying Sun, Rong Bao, Dihan Zhou, Yi Yang, Yuan Cao, Jie Yu, Bali Zhao, Yaoming Li, Huimin Yan, Maohua Zhong

**Affiliations:** 1 Mucosal Immunity Research Group, State Key Laboratory of Virology, Wuhan Institute of Virology, Chinese Academy of Sciences, Wuhan, Hubei 430071 China; 2 Animal Biosafety Level III Laboratory at the Center for Animal Experiment, Wuhan University, Wuhan, Hubei 430071 China

## Abstract

Dental caries is one of the most common global chronic diseases affecting all ages of the population; thus a vaccine against caries is urgently needed. Our previous studies demonstrated that a fusion protein, KF-rPAc, in which rPAc of *S. mutans* is directly fused to the C-terminal of *E. coli*-derived flagellin (KF), could confer high prophylactic and therapeutic efficiency against caries. However, possible side effects, including the high antigenicity of flagellin and possible inflammatory injury induced by flagellin, may restrict its clinical usage. Here, we produced a second-generation flagellin-rPAc fusion protein, KFD2-rPAc, by replacing the main antigenicity region domains D2 and D3 of KF with rPAc. Compared with KF-rPAc, KFD2-rPAc has lower TLR5 agonist efficacy and induces fewer systemic inflammatory responses in mice. After intranasal immunization, KFD2-rPAc induces significantly lower flagellin-specific antibody responses but a comparable level of rPAc-specific antibody responses in mice. More importantly, in rat challenge models, KFD2-rPAc induces a robust rPAc-specific IgA response, and confers efficient prophylactic and therapeutic efficiency against caries as does KF-rPAc, while the flagellin-specific antibody responses are highly reduced. In conclusion, low side effects and high protective efficiency against caries makes the second-generation flagellin-rPAc fusion protein, KFD2-rPAc, a promising vaccine candidate against caries.

## Introduction

Dental caries, one of the most common global chronic diseases distributed unevenly among populations, is still a major oral health problem in most industrialized countries. It affects 60–90% of school-age children and the vast majority of adults^1^, thus an anti-caries vaccine has long been attractive for broad-based dental health in caries prevention^2^, and in the treatment of large infected populations^3^. Dental lesions of caries usually result from the localized dissolution and destruction of teeth^4^ caused primarily by *Streptococcus mutans* (*S. mutans*) infections^5, 6^.

A cell-surface fibrillar protein PAc of *S. mutans*, also designated as antigen I/II, B, or P1, is a main virulence factor that has been implicated in the initial adherence of *S. mutans* to the surface of teeth^7, 8^. At present, PAc has been utilized in different experimental systems and has been proven to be an effective immunogen for caries vaccine development^9–11^. In our previous reports, recombinant flagellin-rPAc fusion protein (KF-rPAc), which consists of an alanine-rich region to proline-rich region (A-P) fragment of PAc from *S. mutans* (rPAc) and flagellin from the *E. coli* K12 strain (KF), was found to be able to induce a robust systemic and mucosal antibody response against rPAc. In the rat model, intranasal immunization of 8.5 μg of KF-rPAc before caries were established could confer a 64.2% prophylactic efficacy^12^. Moreover, intranasal immunization of 8.5 μg of KF-rPAc after caries were established could confer a 53.9% therapeutic effect^13^. Such a low dose makes KF-rPAc an attractive vaccine prototype against caries, the advancement of which is mostly dependent on the development of recombinant flagellin as a robust mucosal adjuvant^14–16^.

Bacterial flagellin is one of a small number of protein pathogen-associated molecular patterns (PAMPs), which can be recognized by cell surface Toll-like receptor 5 (TLR5)^17^ and the cytosolic NOD-like receptor protein 4 (NLRC4) inflammasome receptor NAIP5/NAIP6^18, 19^. Flagellin-mediated activation of TLR5 activates proinflammatory genes including IL-6, TNF-α, KC via MyD88, whereas flagellin-activated NAIP5/6 triggers the assembly of the NLRC4 inflammasome, activation of caspase-1, secretion of IL-1β/IL-18, and pyroptosis of infected cells^20^. The mechanism of flagellin as an adjuvant varied based on the administration route. Flagellin performs its mucosal adjuvant activity dependent on TLR5 activation in respiratory epithelial cells^21, 22^ while through TLR5 and/or NLRC4 activation via systemic administration^23^. For a vaccine to be available for human use, the possible side effects of flagellin including the systemic inflammatory response induced by flagellin and the immunogenicity of flagellin itself should be considered.

Several studies have shown that flagellin triggers a prototypical systemic inflammatory response in mice, including the induction of proinflammatory cytokines and oxidative stress^24–26^. The flagellin–TLR5 axis might also trigger cardiac innate immune responses and result in cardiovascular dysfunction^27^. To balance tolerability and immunogenicity, only doses of 2 or 3 μg per component is favorable^28^. To offer efficient and safe protection, an effort must be made to reduce the inflammatory response but maintain the adjuvanticity induced by flagellin. In another aspect, the very potent immunogenicity of flagellin itself led to a concern that immunity to flagellin might affect the potency of this molecule and induce possible side effects when used as a mucosal adjuvant^29^. Thus, the immunogenicity of flagellin should also be decreased for human use.

The flagellin molecule is composed of highly conserved N/C regions (domains D0/D1) crucial for TLR5 agonist activity and the middle hyper-variable region (domains D2/D3)^30–32^. In our previous studies, we found that chimeric protein KFD-p24 3D, in which the main antigenic and immunogenic regions (domains D2/D3) were replaced with HIV-1 p24, induced lower TLR5 agonist efficacy, fewer proinflammatory responses, and fewer flagellin-specific antibody responses^33^. Moreover, KFD-p24 3D induced a comparable mucosal IgA response as did KF-p24 (p24 directly fused with the full length of flagellin). Based on the flexibility of flagellin, a second-generation flagellin-rPAc fusion protein, KFD2-rPAc, was constructed to reduce the antigenicity of the flagellin part and possible related side effects by replacing the main antigenicity region, the hyper-variable region of KF with rPAc. The resulting chimeric protein, KFD2-rPAc, was comparatively analyzed with KF-rPAc in respect to side effects and protective efficiency against caries.

## Results

### Construction, purification, and characterization of the chimeric protein, KFD2-rPAc

**Figure 1:**
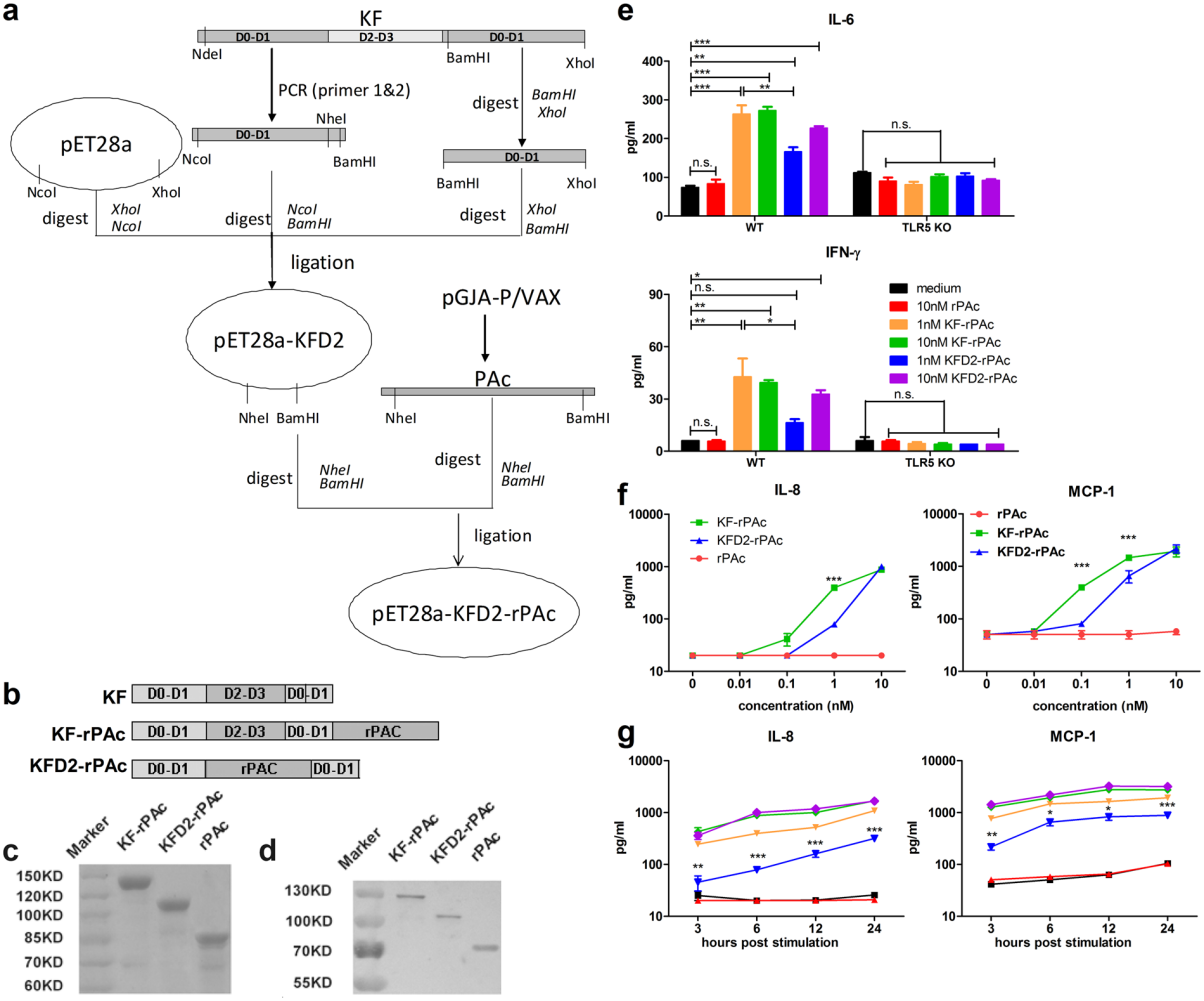
Construction and verification of the recombinant protein KFD2-rPAc. (**a**) Construction process of plasmid pET28a-KFD2-rPAc. (**b**) Diagram of KF, KF-rPAc, and KFD2-rPAc. (**c**) The purity of the recombinant proteins verified by SDS-PAGE. (**d**) Western blotting analysis of the recombinant proteins probed with anti-His tag monoclonal antibody. (**e**) IL-6 and IFN-γ secreted into the culture supernatant from splenocytes of C57BL/6 background WT or TLR5 KO mice stimulated with 10 nM rPAc, 1 or 10 nM KF-rPAc or KFD2-rPAc for 20 hours. Dose-dependent (**f**) and time-dependent (**g**) IL-8 and MCP-1 secreted into the culture supernatant from Caco-2 cells stimulated with indicated concentrations of rPAc, KF-rPAc or KFD2-rPAc. Data are represented as mean ± SEM from triplicate samples of one representative experiment. *p < 0.05; **p < 0.01; and ***p < 0.001; n.s., non-significant.

The expression plasmid pET28a-KFD2-rPAc was constructed by substituting hyper-variable region domains D2 and D3 of flagellin KF with rPAc (Fig. 1a and b) and the recombinant protein was prepared as described in the Materials and Methods section. In the present study, KF-rPAc, KFD2-rPAc, and rPAc in the soluble fraction of cell lysates were purified in parallel. The purified recombinant proteins were tested by SDS-PAGE (Fig. 1c) and Western blotting assay (Fig. 1d). Mice splenocytes from C57BL/6 WT or TLR5 KO mice were used as an *in vitro* model to test the TLR5 agonist efficacy of the recombinant proteins. As shown in Fig. 1e, compared to rPAc or medium alone, both 10 nM of KF-rPAc and KFD2-rPAc induced significantly higher production of IL-6 and IFN-γ from wild type splenocytes but not from TLR5 KO ones. Surprisingly, KFD2-rPAc was less efficient in inducing IL-6 and IFN-γ than KF-rPAc at 1 nM concentration. This demonstrated that KFD2-rPAc has TLR5 agonist activity, but less efficient than its first generation counterpart, KF-rPAc.

To further compare the TLR5 agonist efficiency of KF-rPAc and KFD2-rPAc, extensive assays were carried out on Caco-2 cells, which constitutively express TLR5. At first, the dose-dependent effects of KFD2-rPAc on TLR5 agonist were compared with that of KF-rPAc at 6 hours post stimulation. The results showed that KFD2-rPAc induced IL-8 and MCP-1 in a dose-dependent manner similar to that for KF-rPAc, but with less activity in the concentrations lower than 10 nM (Fig. 1f). To verify this, we further compare the TLR5 activating efficiency between these two recombinants in a wider range of time points during 24 hours post stimulation. Similar to above results, 10 nM of KFD2-rPAc induced comparable IL-8 and MCP-1 production as 10 nM of KF-rPAc, but 1 nM of KFD2-rPAc induced significant less of these soluble mediators than 1 nM of KF-rPAc, at all times points (Fig. 1f).

Besides, the NLRC4 activating efficiency of KF-rPAc, KFD2-rPAc was measured on bone marrow derived macrophages (BMMs). Results showed that similar to KF, KF-rPAc and KFD2-rPAc induced minor production of IL-1β, cell death and caspase-1 p10. Therefore, both KF-rPAc and KFD2-rPAc have poor efficacy in activating the cytosolic NAIP/NLRC4 inflammasome pathway (see Supplementary Fig. S1b–d).

In brief, the second-generation flagellin-rPAc fusion protein, KFD2-rPAc, retained the TLR5 agonist activity but less efficient than KF-rPAc. Moreover, KFD2-rPAc has poor NLRC4 pathway activating efficacy as KF-rPAc and KF.

### KFD2-rPAc induced a much lower systemic inflammatory response than KF-rPAc

Since the TLR5 activating efficiency of KFD2-rPAc is less than that of KF-rPAc, we hypothesized that the TLR5 pathway-related inflammatory responses induced by KFD2-rPAc could be less than that by KF-rPAc. Therefore, we compared potential inflammatory responses and possible side effects induced by KFD2-rPAc and KF-rPAc in the mouse model. PBS and rPAc were used as the vehicle and irrelevant, non-inflammatory protein control, respectively.

**Figure 2:**
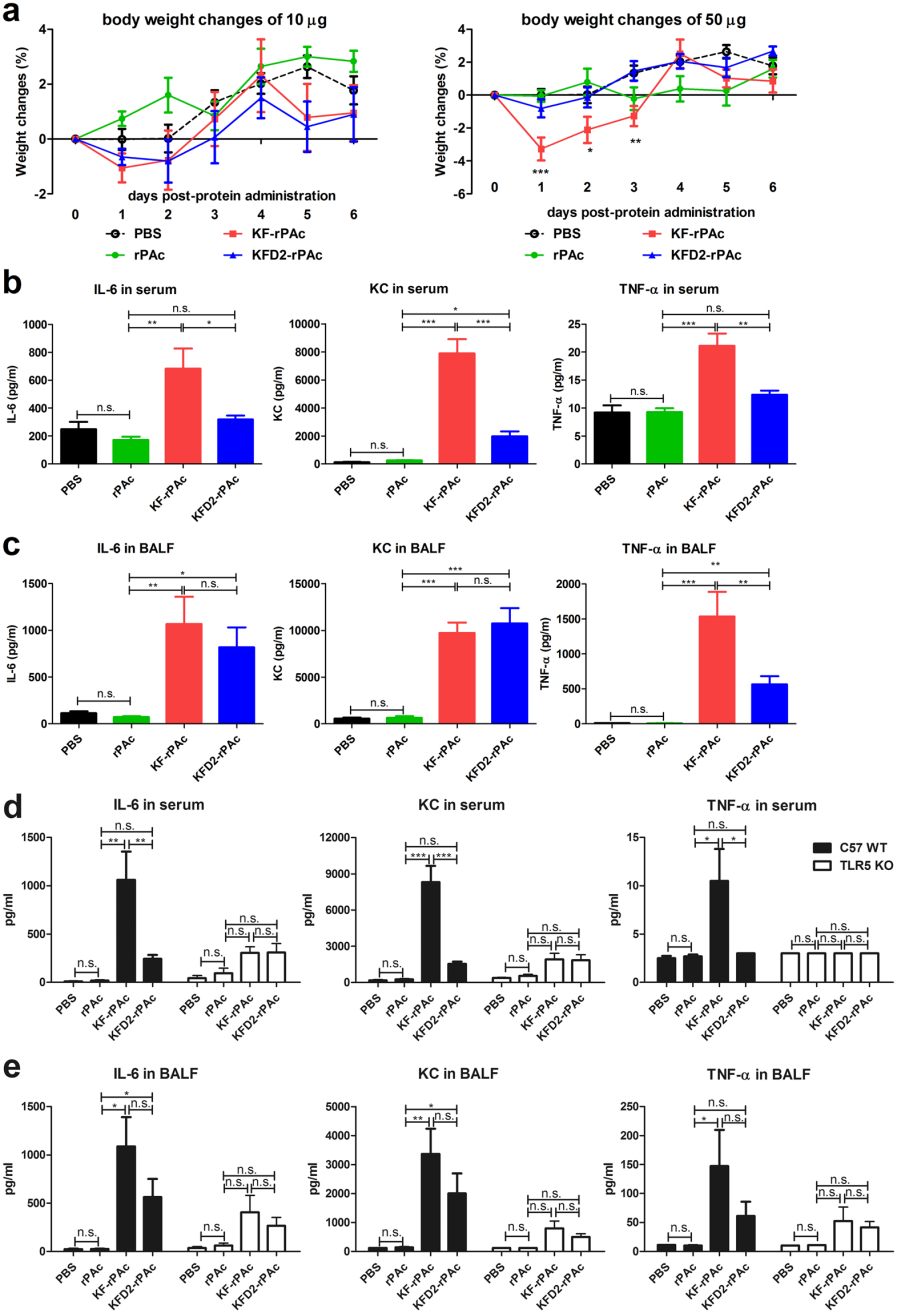
KFD2-rPAc induced a much lower systemic inflammatory response after intranasal administration than KF-rPAc. Female BALB/c or C57BL/6 (WT or TLR5 KO) mice were intranasally treated with indicated amount of rPAc, KF-rPAc, or KFD2-rPAc in 30 μl of PBS or PBS alone. (**a**) Body weight changes of BALB/c mice after 10 μg (left panel) or 50 μg (right panel) recombinant protein administration. IL-6, KC and TNF-α in serum (**b**) or BALF (**c**) after 4 h of administration of 50 μg recombinant protein to BALB/c mice were determined by ELISA kits. IL-6, KC and TNF-α in serum (**d**) or BALF (**e**) after 4 h of administration of 50 μg recombinant protein to C57BL/6 background WT or TLR5 KO mice were determined by ELISA kits. Data are presented as mean ± SEM from 1 experiment that was repeated 3 times (n = 6 per group). *p < 0.05; **p < 0.01; and ***p < 0.001; n.s., non-significant.

First, we analyzed the body weight changes after intranasal administration of 10 μg or 50 μg of KF-rPAc or KFD2-rPAc in BALB/c mice. At the 10-μg dosage, there were no significant body mass changes induced by KF-rPAc or KFD2-rPAc when compared with PBS. However, when the dose was increased to 50 μg, KF-rPAc induced a significant body weight loss at d 1, d 2, and d 3 post-protein administrations. Interestingly, KFD2-rPAc did not induce a significant body weight change during 6 d after protein administration (Fig. 2a).

Next, we analyzed proinflammatory cytokine IL-6, TNF-α and chemokine KC in serum and BALF after intranasal administration of 50 μg of KFD2-rPAc and KF-rPAc^33^. Compared with PBS or rPAc, KF-rPAc induced about a 2-fold increase of IL-6 in the serum, above a 90-fold increase of IL-6 in BALF, over a 30-fold increase of KC in the serum and about a 15-fold increase of KC in BALF. Meanwhile, KF-rPAc induced about a 1-fold increase of TNF-α in the serum and more than a 100-fold increase of TNF-α in BALF. In line with the body weight changes, KFD2-rPAc induced a much lower systemic inflammatory response, which was indicated as significantly lower IL-6, KC and TNF-α induction in serum. Interestingly, KFD2-rPAc also induced much less TNF-α in local fluid, BALF (Fig. 2b and c).

To assess whether KFD2-rPAc-induced less inflammatory responses depend on the activation of flagellin-TLR5 signaling, we performed parallel experiments in C57BL/6 background WT and TLR5 KO mice. As same as in BALB/c mice, C57BL/6 WT and TLR5 KO mice were administrated intranasally with 50 μg rPAc, KF-rPAc and KFD2-rPAc. As shown in Fig. 2d,e, compared to rPAc, KF-rPAc induced significantly increased production of IL-6, KC and TNF-α in serum and BALF, while KFD2-rPAc induced only local increase of IL-6 and KC. On the contrary, neither KF-rPAc nor KFD2-rPAc induced significant increase of IL-6, KC or TNF-α in serum or BALF. These results demonstrated that the inflammatory effects induced by intranasal administration of KF-rPAc and KFD2-rPAc are mainly dependent on TLR5 pathway. And the decreased systemic inflammatory effect presented by KFD2-rPAc is associated with TLR5 pathway.

### KFD2-rPAc induced less flagellin-specific but comparable rPAc-specific antibody responses in mice

**Figure 3:**
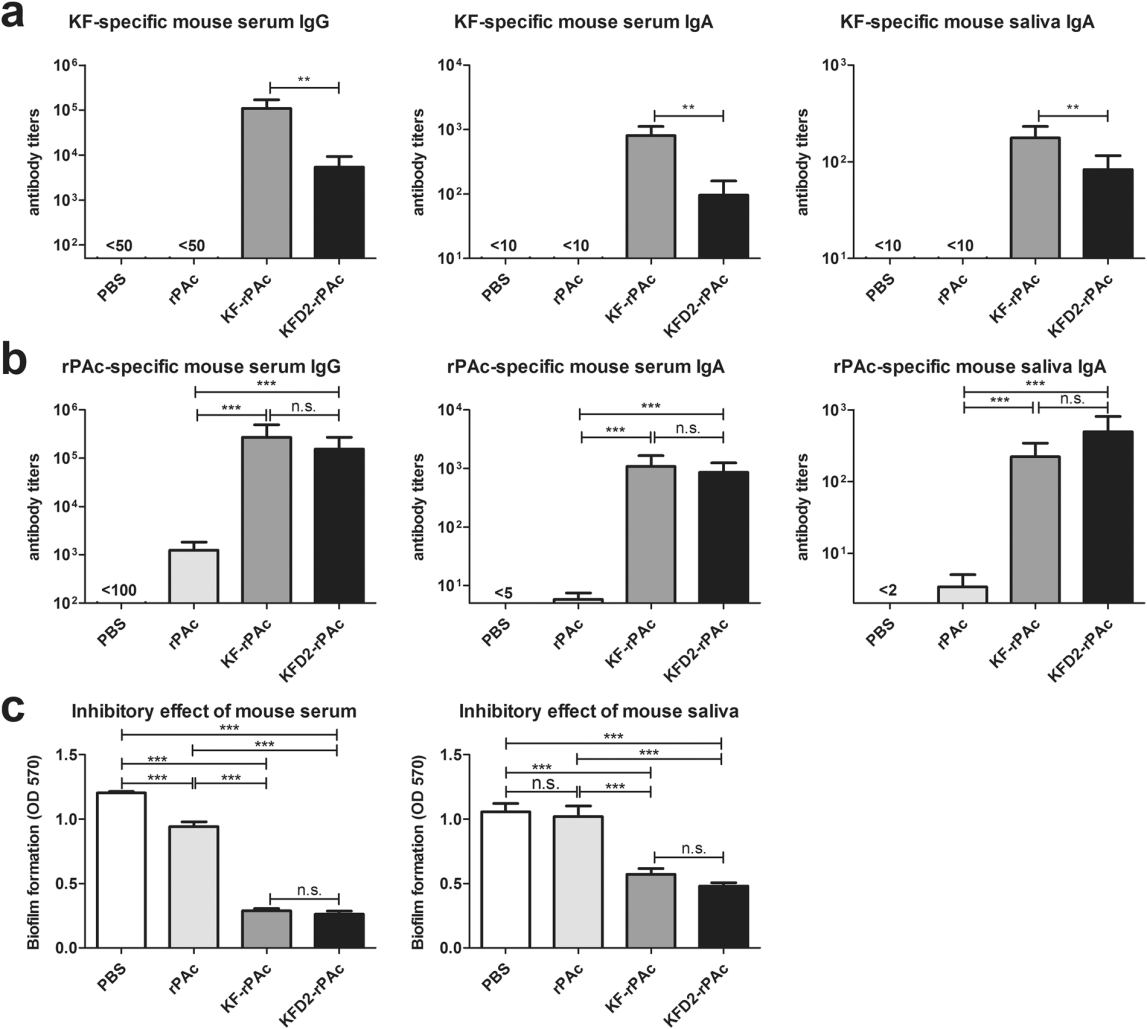
Flagellin- and rPAc-specific antibody responses induced by i.n. immunization of recombinant proteins in mice. Mice were i.n. immunized 3 times at 4-week intervals with PBS, 1 μg of rPAc, 1.7 μg of KF-rPAc, or 1.4 μg of KFD2-rPAc in a 10-μl aliquot. The serum and saliva collected on d 70 were detected by ELISA for antibody response against KF (**a**) or rPAc (**b**). (**a**) KF-specific serum IgG, serum IgA, and salivary IgA. (**b**) rPAc-specific serum IgG, serum IgA, and salivary IgA. (**c**) Biofilm formation inhibition of immunized mice serum or saliva was determined by biofilm assay. Data are represented as mean ± SEM for 6 mice of 1 representative experiment. *p < 0.05; **p < 0.01; and ***p < 0.001; n.s., non-significant.

As the main antigenicity region of flagellin KF, D2 and D3 were replaced with rPAc, and the immunogenicity of the flagellin part in fusion protein KFD2-rPAc would be significantly reduced in our prospects. To comparatively analyze the immunogenicity of KF in KFD2-rPAc and KF-rPAc, mice were intranasally (i.n.) immunized with an equivalent molar amount of protein thrice. Consistent with our hypothesis, KF-specific serum IgG, serum IgA, and salivary IgA induced by KFD2-rPAc were 10 times less than that induced by KF-rPAc (Fig. 3a).

Since the TLR5 activating efficiency of KFD2-rPAc was lower than that of KF-rPAc, we wondered whether the target antigen and rPAc-specific antibody response would be affected. Therefore, the quantity of rPAc-specific antibodies in the serum and saliva was tested and determined by ELISA. Surprisingly, the rPAc-specific serum IgG, serum IgA, and salivary IgA induced by KFD2-rPAc were all on a similar level as that induced by KF-rPAc (Fig. 3b). The antibodies induced by either KFD2-rPAc or KF-rPAc were 100-fold higher than that induced by rPAc alone (Fig. 3b). Then, the quality of the specific antibody responses in the serum and the saliva were tested by an *in vitro* biofilm formation model, which could indicate the inhibitory efficacy of rPAc-specific antibody responses in samples^34^. As shown in Fig. 3c, sera of KFD2-rPAc-immunized mice efficiently inhibited biofilm formation when compared with sera from PBS- or rPAc-immunized mice. More importantly, the inhibiting efficiency of sera from KFD2-rPAc-immunized mice was the same as that from KF-rPAc-immunized mice. In parallel, the saliva of the KFD2-rPAc-immunized group also showed similar efficacy in inhibiting biofilm formation as the saliva of the KF-rPAc-immunized group.

All in all, these results indicated that KFD2-rPAc induces significantly lowered flagellin-specific antibody responses while retaining comparable robust rPAc-specific antibody responses compared with KF-rPAc in mice.

### KFD2-rPAc exhibits high prophylactic efficacy against caries as does KF-rPAc

**Figure 4:**
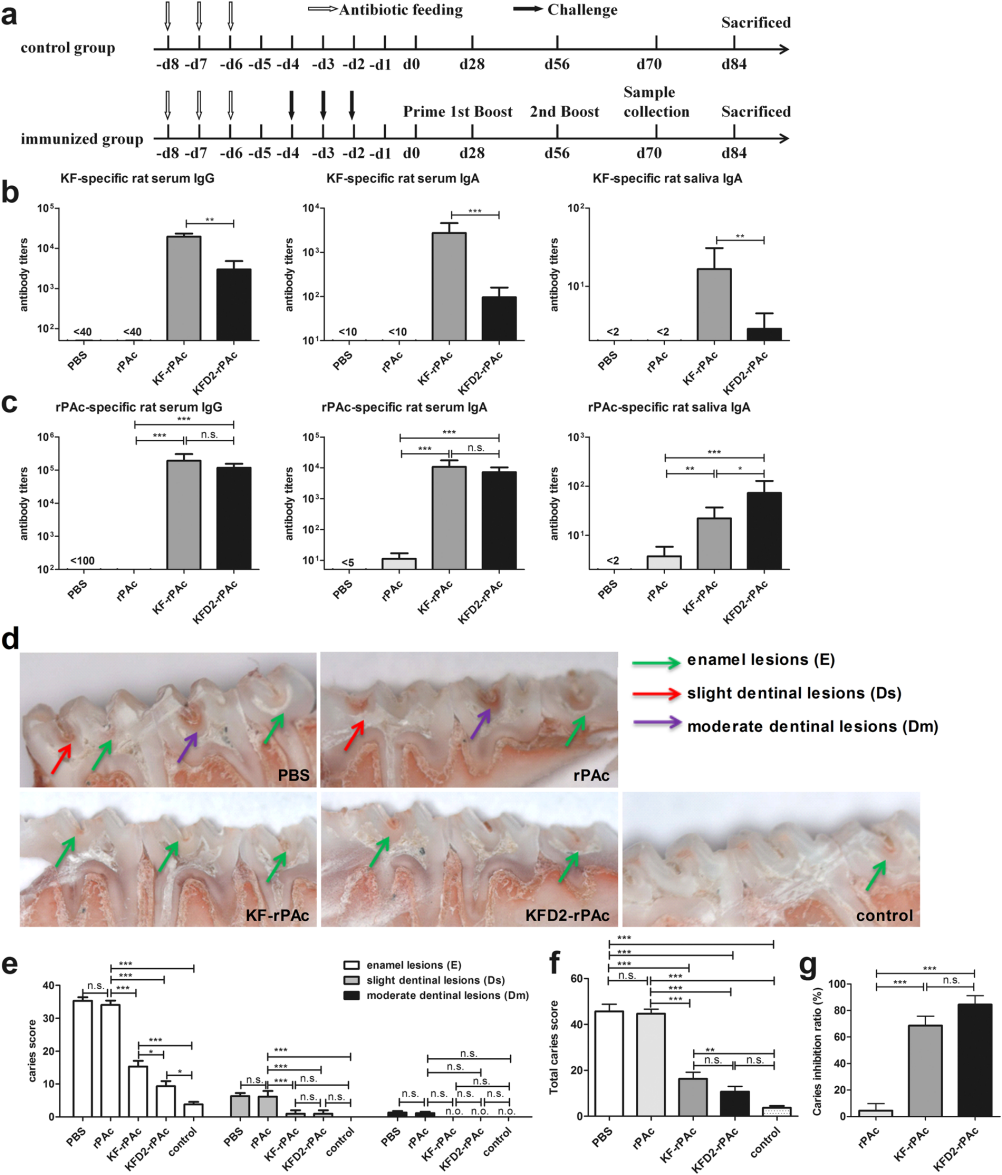
Flagellin- and rPAc-specific antibody responses and prophylactic efficacy of KFD2-rPAc against caries in rats. To analyze the prophylactic efficacy of KFD2-rPAc against caries, rats were immunized before caries were established. The rats were fed antibiotics. After confirming oral bacteria, including *Streptococcus*, were depleted in the oral cavity of the rats, 30 rats were randomly divided into 5 groups (6 rats per group). Four groups of rats were challenged with 2 × 10^9^ CFU of *S. mutans* Ingbritt for 3 consecutive days (once daily), while the other group was left untreated and set as a caries baseline and fed the Keyes 2000 diet. After confirming that all of the challenged rats were successfully infected with bacteria, the rats were immunized with 5 μg of rPAc, 5 µg of rPAc equivalent mole of 8.5 μg KF-rPAc, 7ug KFD2-rPAc, or PBS alone in a 10-μl aliquot, and boosted on d 56 and d 84. The day on which the rats completed the first immunization was set as d 0 (d 0). The serum and the saliva were collected on d 70 and analyzed for flagellin- or rPAc-specific antibody responses by enzyme-linked immunosorbent assay. On d 84, all of the rats were killed and caries levels were scored by the Keyes method. (**a**) Challenge, immunization, and sampling schedule of rats. (**b**) Flagellin-specific serum IgG, serum IgA, and saliva IgA responses in immunized rats. (**c**) rPAc-specific serum IgG, serum IgA, and saliva IgA responses in immunized rats. (**d**) Representative photographs of caries lesion for each group. E, Ds, and Dm lesions are indicated by a green arrow, red arrow, and purple arrow, respectively. (**e**) Caries scores of enamel (E) and slight dentinal (Ds) and moderate dentinal (Dm) lesions of rats immunized with different immunogens. (**f**) Total caries scores of different groups (total score = score of E + Ds + Dm). (**g**) The inhibition ratio of dental caries by immunization with different immunogens. *p < 0.05; **p < 0.01; and ***p < 0.001; n.s., non-significant. n.o., not observed.

Since KFD2-rPAc induces lowered flagellin-specific but comparable rPAc-specific antibody responses in mice, we wondered whether the changed antibody responses by KFD2-rPAc also exist in rats and provide efficient protection against caries or not. First, we analyzed the prophylactic efficacy against caries in the *S. mutans-*challenged rat model based on our previous study^12^, which is briefly depicted in Fig. 4a. Rats were intranasally (i.n.) immunized with an equivalent molar amount of protein thrice. The antibody levels and caries scores were evaluated at the end of the experiments. Similar as observed in the mice, i.n. immunization of KFD2-rPAc induced significantly lower KF-specific serum IgG, serum IgA, and salivary IgA than that of KF-rPAc in the rats (Fig. 4b). Meanwhile, i.n. immunization of KFD2-rPAc induced robust rPAc-specific serum IgG and IgA responses similar to KF-rPAc in the rats, in which both induced about 1000 times higher than that by rPAc alone. Interestingly, it should be noted that i.n. immunization of KFD2-rPAc induced higher rPAc-specific salivary IgA than KF-rPAc (Fig. 4c). Moreover, in KFD2-rPAc-immunized rats, the titer of KF-specific antibody response was over 20 times less than that of the rPAc-specific antibody response (Fig. 4b and c). These results suggested that the replacement of the D2/D3 region of KF with rPAc not only reduces flagellin-specific antibody responses, but it tends to induce more secretory rPAc-specific IgA in saliva.

Corresponding to the antibody responses, significantly fewer caries lesions, including enamel lesions (E) and slight dentinal lesions (Ds), were observed in the rats immunized with the fusion protein KF-rPAc or KFD2-rPAc than in the rats immunized with PBS or rPAc alone. No moderate dentinal lesions (Dm) were observed in the rats immunized with the protein KF-rPAc or KFD2-rPAc. Surprisingly, significantly fewer enamel lesions (E) were observed in the rats immunized with KFD2-rPAc than with the KF-rPAc (Fig. 4d,e). Accordingly, significantly lower total caries scores (E + Ds + Dm) were observed in the rats immunized with protein KF-rPAc or KFD2-rPAc than in the rats immunized with PBS or rPAc alone. Lower total caries scores were shown in the rats immunized with the chimeric KFD2-rPAc than in the rats immunized with KF-rPAc, though not significantly (Fig. 4f). Moreover, among the *S. mutans*-challenged rats, only the KFD2-rPAc-immunized group showed no significant difference in caries lesions from the uninfected rats. Based on the total caries scores of the sham-immunized rats (PBS group, 0%) and the uninfected rats (unchallenged control group, 100%), 84.4% of the mean caries reduction was achieved by i.n. immunization of KFD2-rPAc, 68.5% of the mean caries reduction by KF-rPAc, while only 4.5% was by rPAc alone (Fig. 4g). These results indicated that i.n. immunization of the chimeric KFD2-rPAc greatly prevented teeth against *S. mutans*-induced dental caries in rats.

Together, compared with KF-rPAc, KFD2-rPAc induces less flagellin-specific but comparable rPAc-specific antibody responses in rats and confers comparable prophylactic protection against caries in rats that were immunized before caries were established.

### KFD2-rPAc exhibits a high therapeutic effect against caries as does KF-rPAc

**Figure 5:**
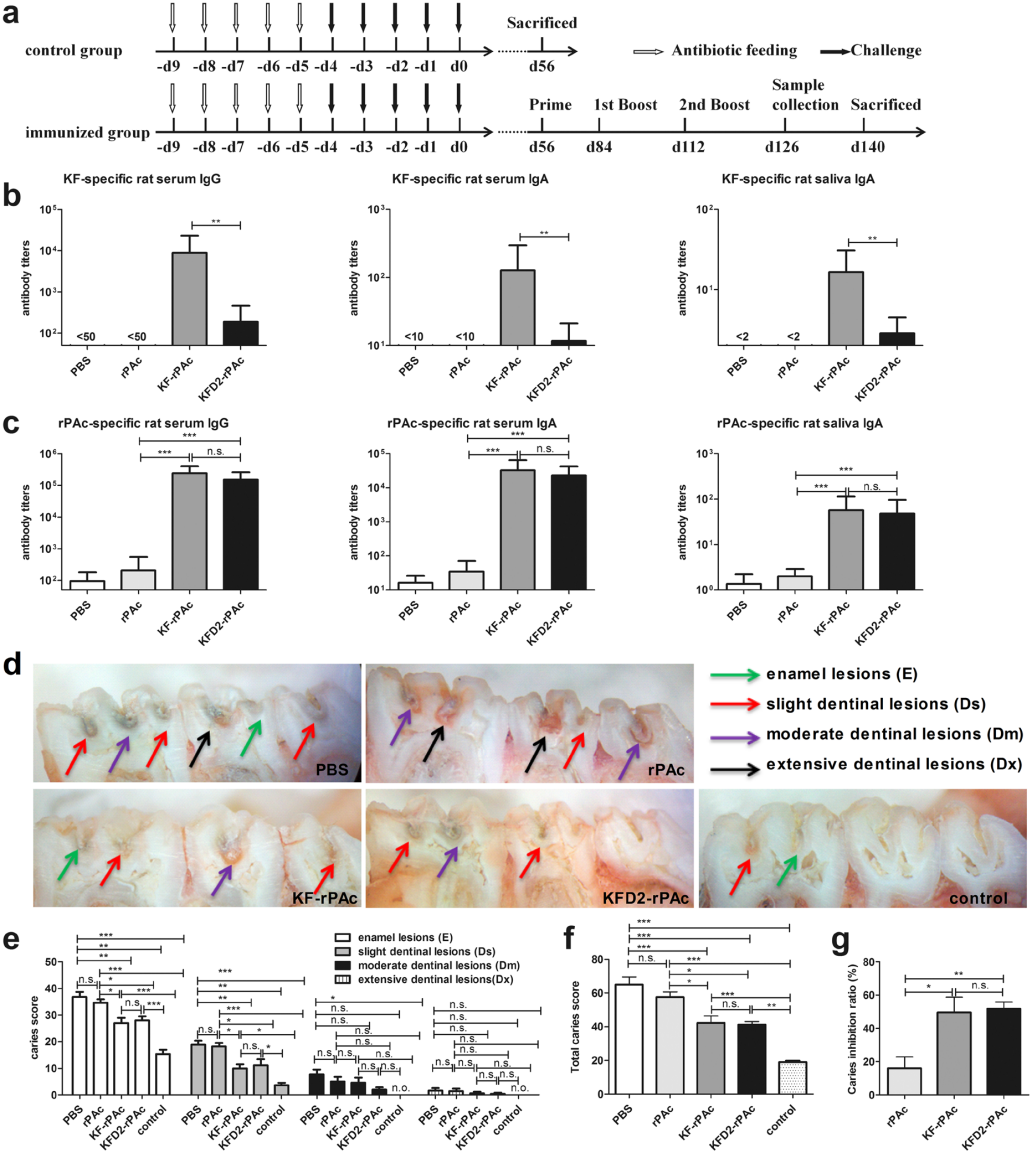
Flagellin- and rPAc-specific antibody responses and therapeutic effect of KFD2-rPAc against caries in rats. To analyze the therapeutic effect of KFD2-rPAc against caries, the rats were immunized after caries were established. Rats were fed antibiotics and challenged with *S. mutans* Ingbritt. The day on which the rats completed the challenge was set as d 0 (d 0). Fifty-six days after implanting *S. mutans*, all of the rats developed E and Ds lesions. Rats were then randomly divided into 5 groups (6 per group): control, PBS, rPAc, KF-rPAc, and KFD2-rPAc. At d 56, the control group was killed and set as the caries baseline, while the other groups were intranasally immunized with PBS, 5 μg of rPAc, 8.5 μg of KF-rPAc, or 7 μg of KFD2-rPAc in a 10-μl aliquot, and boosted on d 84 and d 112, respectively. The serum and the saliva were collected on d 126 and analyzed for flagellin- or rPAc-specific antibody responses by enzyme-linked immunosorbent assay. On d 140, the rats were killed and the caries levels were scored by the Keyes method. (**a**) Challenge, immunization, and sampling schedule of rats. (**b**) Flagellin-specific serum IgG, serum IgA, and saliva IgA responses in immunized rats. (**c**) rPAc-specific serum IgG, serum IgA, and saliva IgA responses in immunized rats. (**d**) Representative photographs of caries lesion for each group. E, Ds, Dm, and Dx lesions are indicated by a green arrow, red arrow, purple arrow, and black arrow, respectively. (**e**) Caries scores of enamel (E), slight dentinal (Ds), moderate dentinal (Dm), and extensive dentinal (Dx) lesions of rats immunized with different immunogens. (**f**) Total caries score of different groups were determined: (total score = score of E + Ds + Dm + Dx). (**g**) The inhibition ratio of dental caries by immunization of different immunogens. *p < 0.05; **p < 0.01; and ***p < 0.001; n.s., non-significant. n.o., not observed.

We further analyzed the therapeutic effect against caries in the *S. mutans-*challenged rat model based on our previous study^13^, which is briefly depicted in Fig. 5a. Eight weeks after implanting *S. mutans*, all of the rats developed E and Ds lesions. Rats were then grouped and intranasally immunized with (1) PBS, (2) 5 μg of rPAc, (3) 8.5 μg of KF-rPAc, or (4) 7 μg of KFD2-rPAc, with the equivalent molar dosage of 5 µg rPAc according to the protocol shown in Fig. 5a. After the second boost, KF-specific serum IgG, serum IgA, and salivary IgA induced by KFD2-rPAc were about 10 times less than that elicited by KF-rPAc (*p* < 0.05) (Fig. 5b). For the rPAc-specific response, both KFD2-rPAc and KF-rPAc induced about 1000-fold higher rPAc-specific serum IgG, 200-fold higher rPAc-specific serum IgA, and 20-fold higher salivary IgA responses than that induced by rPAc alone. Compared with the KF-rPAc immunization, the KFD2-rPAc immunization induced comparable rPAc-specific serum IgG, serum IgA, and salivary IgA (Fig. 5c). Moreover, in the KFD2-rPAc-immunized rats, the titer of KF-specific antibody response was more than 50 times less than that of the rPAc-specific antibody response (Fig. 5b and c).

After rats were killed at 4 weeks post-final immunization, the caries scores were detected for each rat subjected to the experiments. As shown in Fig. 5d, significantly fewer E and Ds lesions were observed in the rats immunized with KF-rPAc or KFD2-rPAc than those immunized with PBS or rPAc alone (Fig. 5d,e). Accordingly, significantly lower total caries scores were also observed in the rats immunized with KF-rPAc or KFD2-rPAc than those immunized with PBS and rPAc (Fig. 5f). In comparison with the sham-immunized rats (PBS group, 0%) and the unchallenged control group (killed at 8 weeks post-bacterial challenge, 100%), the rats immunized with KF-rPAc or KFD2-rPAc exhibited similar caries reductions (49.6% vs 51.7%), both of which were significantly higher than that of rPAc groups (Fig. 5g).

## Discussion

In this study, we characterized the second-generation flagellin-rPAc fusion protein, a vaccine candidate designed to avoid an undesired flagellin-specific antibody response and inflammatory side effects while inducing efficacious antibodies against PAc and providing high protective efficacy against dental caries.

Many studies have addressed active immunity for dental caries in rat models. The induction of salivary IgA and serum IgG has been studied using various immunization routes together with various adjuvants and delivery vehicles in rats^35^. As early as 1993, *S. mutans* Antigen I/II coupled to the B subunit of cholera toxin (CTB) was tested by intranasal immunization in conventional rats, which achieved an efficacy of 38% reduction of *S. mutans* in plaque and a 64% reduction in buccal enamel caries with 3 doses of 50 μg of protein^36^. A recent study reported that i.n. immunization with 3 doses of 50 μg of plasmid pGJA-P/VAX-bupivacaine complexes in rats was able to mount the specific salivary IgA response and confer about a 60% reduction in dental caries lesions^37^. When 100 μg of plasmid pGJA-P/VAX was i.n. immunized together with 20 μg of recombinant flagellin as an adjuvant in rats, a 47% caries reduction could be achieved^38^. However, the inherent toxicity of CTB and the high cost for DNA vaccine production preclude the use of the above-mentioned vaccines in humans. Taking the recombinant flagellin as an effective and feasible adjuvant into account^15^, we constructed a single recombinant fusion protein, KF-rPAc, in which rPAc of *S. mutans* is fused to the C-terminal of flagellin KF, and after i.n. immunization with only a 8.5-μg dose, it afforded a 64.2% caries reduction in rats before the development of caries^12^ and conferred a 53.9% protection efficacy in terminating the progression of established caries^13^. Meanwhile, the adjuvant KF or target antigen rPAc alone didn’t induce any significant protection against caries in immunized rats^12^. The above data demonstrate that the flagellin-rPAc fusion protein is an attractive caries vaccine candidate.

The protein property of flagellin confers it with both the advantage of easy modification and the disadvantage of immunogenicity. Flagellin contains two highly conserved N/C domains (D0 and D1) and one central hypervariable domain (D2/D3). The conserved D0 and D1 domains are required for the immune activity of flagellin as a pathogen-associated molecular pattern^39–41^. D1 interacts directly with TLR5^42^, and the N-terminal amino acid residues 90–97 (QRVRELAV) of D1 form a highly conserved motif that is essential for both high-affinity binding to TLR5 and subsequent signaling^43^. The hypervariable domain D2/D3 is vastly diverse in size and amino-acid composition among bacterial strains and species, and about 90% antigenicity of flagellin were located in this domain^33^. This hypervariable domain D2/D3 promotes TLR5 domain-specific neutralization of antibody production, which robustly impairs TLR5 activating efficiency of flagellin^29^. Moreover, D2/D3 probably directly interferes with the adaptive immune response against foreign antigens by competing the innate soluble factors and cells because of its high immunogenicity.

To reduce the immunogenicity of flagellin, we constructed a second-generation flagellin-rPAc fusion protein, KFD2-rPAc, in which rPAc replaced D2/D3, the main antigenicity domains of KF. Results in this study demonstrated that the immunogenicity of flagellin itself is substantially reduced in KFD2-rPAc. KFD2-rPAc induced over 10-fold less flagellin-specific antibody responses in mice and rats (Figs 3a, 4b and 5b). The significantly lowered immunogenicity of flagellin partly makes KFD2-rPAc more feasible for multiple administrations without interference by pre-existed antibodies.

In developing a vaccine for human use, safety concerns are even more important. In addition to the immunogenicity of flagellin being restricted, the potential inflammatory response induced by flagellin should also be restricted. The flagellin/TLR5 axis-induced response is a double-edged sword for its adjuvanticity and side effects. On one hand, flagellin exerts its adjuvanticity by activating a range of innate immune cells secreting certain cytokines and chemokines, which trigger an adaptive immune response. On the other hand, flagellin triggers a prototypical systemic inflammatory response, including the induction and secretion of proinflammatory cytokines in the lungs, small intestine, liver, cardiovascular system, and kidneys^27, 44^.

Therefore, to balance TLR5-associated adjuvanticity and the potential inflammatory response, an ideal vaccine candidate should contain moderate TLR5 activating efficiency. Compared with KF-rPAc, KFD2-rPAc has lowered TLR5 agonist activity (Fig. 1e–g). To explore the potential side effects, up to 50 μg of KFD2-rPAc and KF-rPAc was instilled into the lungs of mice. The reduced TLR5 agonist activity led to a reduced inflammatory response, which was demonstrated as less proinflammatory cytokine IL-6, chemokine KC (also designated as CXCL1), in the serum, and less proinflammatory TNF-α in the serum and local fluid BALF, and diminished body weight loss (Fig. 2). There are extensive literatures showing that IL-6 plays an important role in the initiation and development of inflammatory responses, including recruitment and apoptosis of leukocytes, maintenance of the effector function of T cells, and the inflammatory activation of stromal tissues^45^. KC plays a critical role in the recruitment and activation of polymorphonuclear cells, especially neutrophils to inflammatory sites during several inflammatory processes^26, 46^. The recruited neutrophils are active, resulting in the formation of a phagosome and a respiratory burst^26^. TNF-α is a proinflammatory and immune-regulatory cytokine that enhances leukocyte migration, promotes the transcription of several inflammatory genes, and causes apoptosis of epithelial cells^47^. In addition, anti-TNF-α is widely used as a strategy to treat many kinds of inflammatory diseases. Therefore, robust IL-6, TNF-α and KC induction represent the potential proinflammatory response of the flagellin-rPAc fusion protein. Consistent with reduced IL-6, TNF-α and KC induction, body weight changes induced by KFD2-rPAc were also reduced. Based on our results, the second-generation flagellin-rPAc fusion protein, KFD2-rPAc, showed much lower potential side effects and is much safer than the first-generation fusion protein, KF-rPAc.

As for the reason that KFD2-rPAc induced a different profile of IL-6, KC and TNF-α reduction in BALF when compared with KFD2-rPAc, we suggest cell origin differences. IL-6 can be produced by almost every immune cells and many non-immune cells including endothelial cells, fibroblasts and keratinocytes^45^, and KC is produced mainly by epithelial cells and endothelial cells^48, 49^, while TNF-α is secreted by immune cells, including macrophages, monocytes, neutrophils, T-cells (principally CD4+), and NK-cells^50^. Moreover, there are big differences in TLR5 expression levels among different types of cells. Both Lung epithelial cells and endothelial cells express TLR5 at a considerable level^21, 51^, but immune cells express minimal TLR5^52, 53^. Although KFD2-rPAc induced a significantly lower level of IL-8 (Fig. 1e) than KF-rPAc at the 1-nM level, at a high dosage up to 50 μg, the TLR5 responding efficiency of lung epithelial and endothelial cells may be saturated, so KFD2-rPAc and KF-rPAc induced similar production of IL-6 and KC in BALF. However, because of the ultra-low level of TLR5 expression, immune cells were not so sensitive to the flagellin-associated protein and the difference in TLR5 activating efficiency between KFD2-rPAc and KF-rPAc was still shown.

After intranasal administration, flagellin is mainly restrained to the conducting airways, but very small amounts could be translocated into the parenchymal compartment of the lungs, blood, or peripheral tissues^21^. This minimal translocation of KFD2-rPAc and KF-rPAc to blood can be convinced by the ultra-low production of TNF-α in serum, less than 25 pg/ml versus 1500 pg/ml in BALF (for BALB/c mice, Fig. 2b,c). Therefore, the concentration of KFD2-rPAc and KF-rPAc in blood is much lower than that in lungs. It can be speculated that KFD2-rPAc and KF-rPAc are in blood, activating blood vessel endothelial cells to produce IL-6 and KC. Accordingly, the TLR5 activating efficiency differences between KFD2-rPAc and KF-rPAc can be seen as serum IL-6 and KC induced by KFD2-rPAc as being much less than that by KF-rPAc. In parallel, KFD2-rPAc also induced less TNF-α than KF-rPAc.

Interestingly, KFD2-rPAc retains the ability to induce robust rPAc-specific antibody responses in mice and in rats (Figs 3b, 4c and 5c). Accordingly, KFD2-rPAc induced comparable immune protection (Figs 3c, 4d–g and 5d–g). We speculated that although there was reduced TLR5 activating efficiency, the rPAc-specific antibody response induced by KFD2-rPAc caught up with that by KF-rPAc after 3 i.n. immunizations.

In conclusion, KFD2-rPAc, the second-generation flagellin-rPAc fusion protein, induced low potential systemic inflammatory responses and low flagellin-specific antibody responses, but high immune protection against caries. These advantages make KFD2-rPAc a promising anti-caries vaccine candidate.

## Materials and Methods

### Mice and rats

Female BALB/c and C57BL/6 mice, aged 6–8 weeks were obtained from Beijing Laboratory Animal Research Center and housed in the Animal Center of Wuhan Institute of Virology (WIV), Chinese Academy of Sciences (CAS), under specific pathogen-free (SPF) conditions. C57BL/6 background Tlr5 knockout mice (TLR5 KO) of Jackson laboratory origin were bred and housed in the Animal Center of Wuhan Institute of Virology (WIV), Chinese Academy of Sciences (CAS) under specific pathogen-free (SPF) conditions. SPF 18-day-old female weanling Wistar rats were purchased from Hubei CDC (Wuhan, China). Animal studies were performed according to the Regulations for the Administration of Affairs Concerning Experimental Animals in China (1988) and the Guidelines for Animal Care and Use, WIV, CAS. Animal experiments were reviewed and approved by Institutional Review Board (IRB), WIV, CAS (permission number: WIVA09201211). All animals were randomly assigned to groups before the experiments were performed.

### Construction of chimeric protein expression plasmid and protein purification

The *fliC* gene from the *E. coli* K12 strain MG1655 (GenBank Accession No. 949101) and the A-P fragment, from amino acid residues 219 to 905 of the PAc protein encoded by the *pac* gene of *S. mutans* MT8148, were obtained from previous research^54^. The truncated *fliC* gene, kfd2, was from our previous study^33^. The fragments were cloned into the pET28a plasmid vector (Invitrogen, ThermoFisher Scientific, USA) to construct the expression plasmids of pET28a-KF, pET28a-rPAc, pET28a-KF-rPAc, and pET28a-KFD2-rPAc (Fig. 1). All of the expression plasmids were transformed into competent *E.coli* BL21 (DE3), and verified by DNA sequencing (Invitrogen).

Recombinant flagellin (designated as KF), PAc (designated as rPAc), and flagellin-rPAc fusion proteins (KF-rPAc and KFD2-rPAc) were purified by affinity chromatography on a Ni-NTA column (Qiagen, Hilden, Germany) and dialyzed with PBS at 4 °C as previously described^14^. Residual LPS was removed as previously described^55^. Concentrations of the purified proteins were detected by Bradford protein assay^56^. The purity of proteins was assessed by SDS-PAGE and Western blotting with an anti-His Tag monoclonal antibody (Beyotime Biotechnology, Shanghai, China**)** and a secondary goat anti-mouse IgG conjugated to alkaline phosphatase (SouthernBiotech, Birmingham,AL USA). Residual endotoxin content was determined with the Pierce LAL chromogenic endotoxin quantitation kit (ThermoFisher Scientific, USA). Endotoxin values of recombinant proteins for immunization were <0.005 EU/μg. RAW 264.7 cells, which could sensitively respond to lipopolysaccharide (LPS) and bacterial DNA but not to flagellin^57, 58^, were used to exclude the presence of residual bacterial DNA and LPS contamination (see Supplementary Fig. S1a).

### Cytokine detection for *in vitro* bioassay of TLR5-specific signaling

Caco-2 cells were maintained in the Dulbecco modified Eagle medium (Invitrogen) supplemented with 10% fetal bovine serum (Gibco, ThermoFisher Scientific, USA). The cells were seeded 2 × 10^5^/well in 24-well plates and maintained at 37 °C in 5% CO_2_ for 5 d to form a tight monolayer and then they were cultured in DMEM without serum overnight, and subsequently stimulated with proteins at serial concentrations (0.01 to 10 nM). The supernatants were collected at the indicated time points for detection of IL-8 and MCP-1 by enzyme-linked immunosorbent assay (ELISA) kits (BD bioscience).

Spleens obtained from 6–8 weeks old female C57BL/6 mice (wild type (WT) or TLR5 KO) were smashed using syringe pistons in PBS and filtered through strainers (BD Biosciences). After washing with PBS and RPMI 1640 medium containing 10% FBS and 100 U/ml of penicillin/streptomycin, single cells were seeded into 48-well plates (4 × 10^6^ cells/well, 0.2 ml) in medium. Cells were stimulated with 1 or 10 nM rPAc, KF-rPAc or KFD2-rPAc. The supernatants were collected at 20 h post stimulation to assess cytokines IL-6 and IFN-γ by ELISA kits (BioLegend, USA).

### NLRC4 pathway activation efficiency detection in bone marrow-derived macrophages (BMMs)

NLRC4 pathway activation efficiency of recombinant proteins was detected in BMMs as previously described^55^. Briefly, BMMs were pretreated with LPS at a concentration of 50 ng/ml for 3 h and then transfected with proteins using DOTAP (Roche, Basel, Switzerland). Supernatants were collected at 20 h after transfection for IL-1β detection by ELISA (BioLegend, USA). Cell death was detected by the lactate dehydrogenase (LDH) release assay with the Cytotoxicity Detection Kit^PLUS^ (Roche). Caspase-1 p10 levels were quantified by Western blotting assay with specific antibody (Santa Cruz Biotechnology, USA).

### Body weight changes

Female BALB/c mice were anesthetized and intranasally administrated 10 μg or 50 μg of rPAc, KF-rPAc, KFD2-rPAc, or PBS alone in 30-μl volume. The body weight of each mouse was observed immediately before intranasal immunization and every 24 h after intranasal administration for 6 d.

### Cytokine determination in serum and bronchoalveolar lavage fluid (BALF)

Female BALB/c mice or C57BL/6 background WT or TLR5 KO mice were anesthetized and intranasally administrated 50 μg of rPAc, KF-rPAc, KFD2-rPAc, or PBS alone in 30-μl volume. The animals were killed by cervical dislocation at 4 h post administration. Serum and bronchoalveolar lavage fluid (BALF) were collected as described previously^59^. Bronchoalveolar lavage (BAL) was performed by the intratracheal instillation of 1 mL of PBS, reinfused 8 times into the lung. The BALF was centrifuged, and the cell-free supernatant was frozen at −70 °C until ELISA. Proinflammatory cytokine IL-6, TNF-α and chemokine KC (also designated as CXCL1) in serum and BALF were then quantified by ELISA kits (IL-6 and TNF-α ELISA kits from BioLegend, USA and KC ELISA kits from Multi Sciences Biotech, China) according to the manufacturers’ instructions.

### Mice immunization and sample collection

Female BALB/c mice were randomly divided into 4 groups (6 per group), and intranasally immunized with 1 μg of rPAc, 1.7 μg of KF-rPAc, 1.4 μg of KFD2-rPAc or PBS alone, respectively, in a 10-μl aliquot. The immunization was performed 3 times as primed on d 0, and boosted twice on d 28 and d 56. Serum and saliva samples were collected on d 70 and analyzed for KF- and rPAc-specific antibody responses by ELISA as described previously^33^. The biofilm formation inhibition assay was carried out to check the quality of rPAc-specific antibody response in the serum and saliva of immunized mice by biofilm assay as previously described^34^.

### Rat immunization, antibody analysis, and caries model

The efficacy of KFD2-rPAc against caries was evaluated by two protocols of rat models: Protocol A was used to test prophylactic efficacy (immunization was carried out before caries developed) and Protocol B was used to test the therapeutic effect (immunization was carried out after caries were established)^12, 13^.

Protocol A, which was used to evaluate the prophylactic efficacy of KFD2-rPAc before caries developed, is illustrated in Fig. 4a as described previously^12^. Briefly, rats were weaned and raised on the Keyes 2000 cariogenic diet. Rats were fed antibiotics for 3 consecutive days. Then, 24 h later, using swabs pre-soaked with physiological saline and cultured on solid MSB medium (Becton Dickinson, San Jose, CA, USA), bacterial samples from occlusal surfaces of each rat were examined to ensure that oral bacteria, including Streptococcus, were depleted in the oral cavity. Rats were then randomly divided into 5 groups (6 per group). Four groups of rats were challenged with 2 × 10^9^ CFU of *S. mutans* Ingbritt for 3 consecutive days (once daily), while the other group was left untreated to establish a caries baseline and they were fed the Keyes 2000 diet. After confirming that all of the challenged rats were successfully infected with bacteria, the rats were immunized with 5 μg of rPAc, 5 µg of rPAc equivalent mole of 8.5 μg of KF-rPAc, 7 μg of KFD2-rPAc, or PBS alone in a 10-μl aliquot, and boosted on d 56 and d 84. The day on which the rats completed the first immunization was set as d 0 (d 0), and the rats were 28-days-old at d 0. Serum and saliva were collected on d 70 and analyzed for antibody responses by ELISA as previously described^12^. On d 84, all of the 30 rats were killed. The teeth were hemisected and observed by a stereomicroscope (Zeiss, Jena, Germany), and caries levels were scored by the Keyes method^60^. The inhibition ratio was calculated as: [1 − (Total Score_experimental group_ − Total Score_control group_)/(Total Score_PBS group_ − Total Score_control group_)] × 100%. All of the experiments were repeated 3 times.

Protocol B, which was used to evaluate the therapeutic effect after caries developed, is shown in Fig. 5a as described previously^13^. Briefly, rats were weaned and raised on the Keyes 2000 cariogenic diet. Rats were fed antibiotics for 5 consecutive days, and then rats were challenged by 2 × 10^9^ CFU of *S. mutans* Ingbritt 5 times (once daily). The day on which the rats completed the challenge was set as d 0 (d 0), and the rats were 28-days-old at d 0. Fifty-six days after implanting *S. mutans*, all of the rats developed E and Ds lesions. Rats were then randomly divided into 5 groups (6 per group): control, PBS, rPAc, KF-rPAc, and KFD2-rPAc. At d 56, the control group was killed and set as the caries baseline, while the other groups were intranasally immunized with PBS, 5 μg of rPAc, 8.5 μg of KF-rPAc, or 7 μg of KFD2-rPAc in a 10-μl aliquot, and boosted on d 84 and d 112. Serum and saliva were collected on d 126 and analyzed for antibody responses by ELISA as previously described^13^. On d 140, the rats were killed. The teeth were hemisected and observed by a stereomicroscope (Zeiss, Jena, Germany), and caries level were scored with the Keyes method^60^. The inhibition ratio was calculated as: [1 − (Total Score_experimental group_ − Total Score_control group_)/(Total Score_PBS group_ − Total Score_control group_)] × 100%. All of the experiments were repeated 3 times.

### Statistics

Data were analyzed by using GraphPad Prism software (San Diego, CA 5. All of the data analysis was performed with one-way ANOVA. When the *p* value was significant at the 5% level, further pair-wise comparisons were made between the experimental group and control conditions using Tukey’s multiple comparisons test. For the statistical analysis of antibody titers, the titers were first transformed to log10.

## Electronic supplementary material

Supplementary Information
