# Supplementary material for: Second-generation Flagellin-rPAc Fusion Protein, KFD2-rPAc, Shows High Protective Efficacy against Dental Caries with Low Potential Side Effects
Source: Sci Rep. 2017 Sep 11;7:11191. doi: 10.1038/s41598-017-10247-8 (PMC5593867; doi:10.1038/s41598-017-10247-8)
Supplement: Supplementary file 1 — Supplementary Information [file 41598_2017_10247_MOESM1_ESM.doc]

**Second-generation Flagellin-rPAc Fusion Protein, KFD2-rPAc, Shows High Protective Efficacy against Dental Caries with Low Potential Side Effects**

Jingyi Yang^1^, Ying Sun^1^, Rong Bao^1,2^, Dihan Zhou^1^, Yi Yang^1^, Yuan Cao^1^, Jie Yu^1^, Bali Zhao^1^, Yaoming Li^1^, Huimin Yan^1^, Maohua Zhong^1*^

^1^Mucosal Immunity Research Group, State Key Laboratory of Virology, Wuhan Institute of Virology, Chinese Academy of Sciences, Wuhan, Hubei 430071, China;

^2^Animal Biosafety Level III Laboratory at the Center for Animal Experiment, Wuhan University, Wuhan, Hubei 430071, China


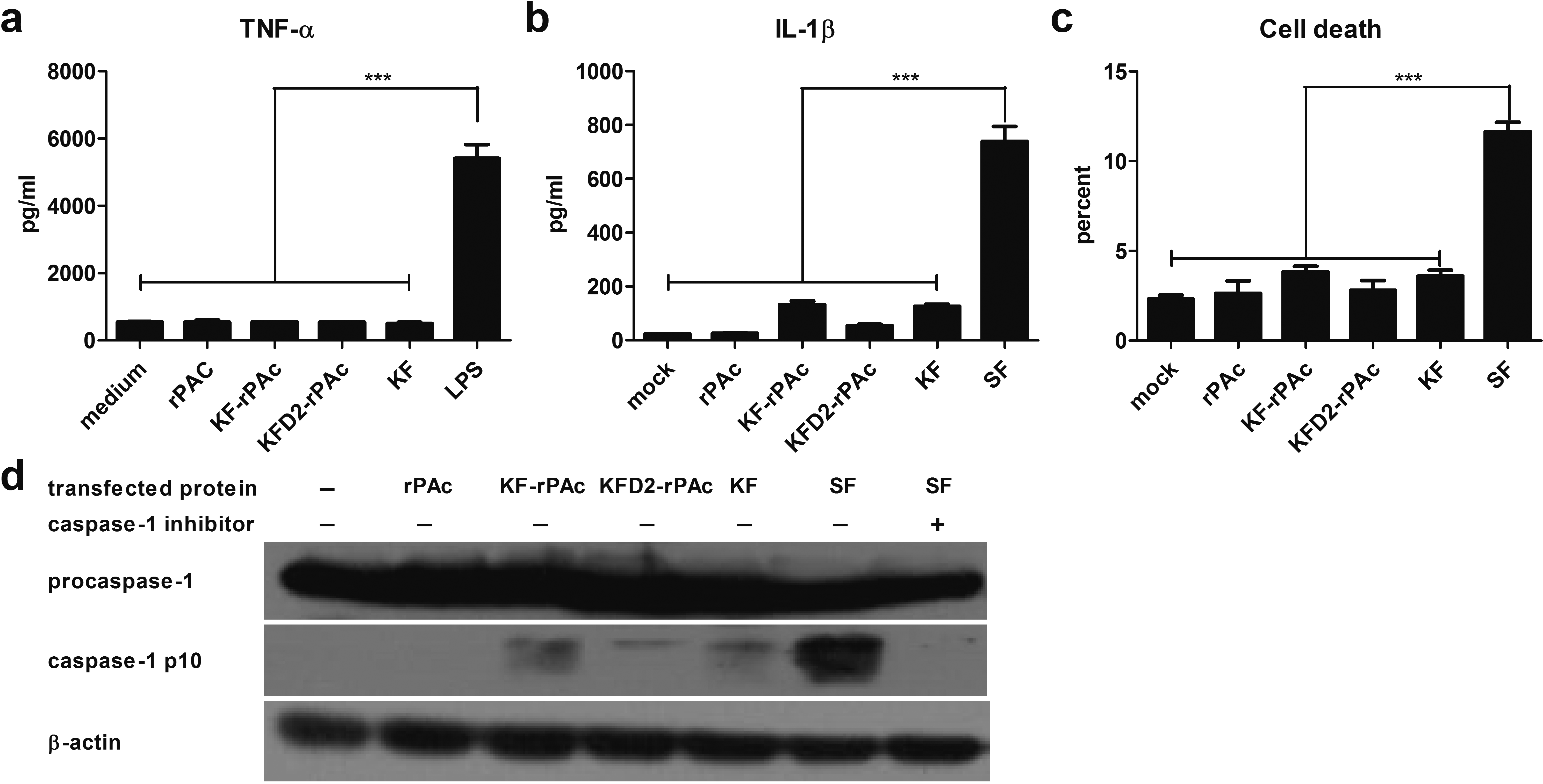


**Fig. S1 Characterization of recombinant proteins.** (a) Detection of the DNA and LPS residue contamination in recombinant proteins with RAW264.7 cells. RAW264.7 cells were seeded into 96-well plates (1.2×10^5^ cells/well) in 0.2 ml DMEM medium containing 10% FBS and 100 U/ml of penicillin/streptomycin overnight, and then stimulated by medium containing 100nM recombinant proteins or 50 ng/ml LPS for 20h. TNF-α in supernatants was detected by ELISA. (b-d) NLRC4 pathway activation efficiency of recombinant proteins was detected in bone marrow-derived macrophages (BMMs). BMMs were pretreated with LPS at a concentration of 50 ng/ml for 3 h and then transfected with proteins using DOTAP. Supernatants were collected at 20 h after transfection for IL-1β detection by ELISA (b). Cell death was detected by the lactate dehydrogenase (LDH) release assay (c). Caspase-1 p10 levels were quantified at 1 h after transfection by Western blotting assay with specific antibody (d).
